# Autophagy dark genes: Can we find them with machine learning?

**DOI:** 10.1002/ntls.20220067

**Published:** 2023-05-01

**Authors:** Mohsen Ranjbar, Jeremy J. Yang, Praveen Kumar, Daniel R. Byrd, Elaine L. Bearer, Tudor I. Oprea

**Affiliations:** 1 Department of Chemistry and Chemical Biology, University of New Mexico, Albuquerque, New Mexico, USA; 2 Alzheimer’s Disease Research Center, University of New Mexico, Albuquerque, New Mexico, USA; 3 Department of Pathology, University of New Mexico Health Sciences Center, Albuquerque, New Mexico, USA; 4 Department of Internal Medicine, University of New Mexico School of Medicine, Albuquerque, New Mexico, USA; 5 Department of Computer Science, University of New Mexico, Albuquerque, New Mexico, USA; 6 Autophagy Inflammation and Metabolism Center of Biomedical Research Excellence, University of New Mexico Health Sciences Center, Albuquerque, New Mexico, USA

**Keywords:** autophagy genes, extreme gradient boosting (XGBoost), knowledge-graph, machine learning, mining functional genomic data

## Abstract

Identifying novel autophagy (ATG) associated genes in humans remains an important task for understanding this fundamental physiological process. Machine learning (ML) can highlight potentially “missing pieces” linking core ATG genes with understudied, “dark” genes by mining functional genomic data. Here, a set of 103 (out of 288 genes from the Autophagy Database) was used as training set, based on ATG-associated terms annotated from 3 secondary sources: GO (gene ontology), Kyoto Encyclopedia of Genes and Genomes pathway, and UniProt keywords, as additional confirmation of their importance in ATG. As negative labels, an OMIM list of genes associated with monogenic diseases was used (after excluding the 288 ATG-associated genes). Data related to these genes from 17 different sources were compiled and used to derive a trained MetaPath/XGBoost (MPxgb) ML model for distinguishing ATG and non-ATG genes (10-fold cross-validated, 100-times randomized models, median area under the curve = 0.994 ± 0.008). Sixteen ATG-relevant variables explained 64% of the total model gain. Overall, 23% of the top 251 predicted genes are annotated in the Autophagy Database, whereas 193 genes (77%) are not. In 2019, we suggested that some of these 193 genes may represent “ATG dark genes.” A literature search in 2022 for those top 20 predicted ATG dark genes found that 9 were subsequently reported as ATG genes during the intervening 3.5 years. A post-factum evaluation of data leakage (the presence of ATG-associated terms in the top 40 ML features) confirms that 7 out of these 9 genes and 2 out of 3 other recently validated predictions from the bottom 20 are novel. Those genes with the largest number of ATG features would be most likely to yield valuable experimental insights. Modern high-throughput testing would be capable of spanning the full 193 ATG genes list reported here. Our analysis demonstrates that ML can guide genomics research to gain a more complete functional and pathway annotation of complex processes.

## Graphical Abstract

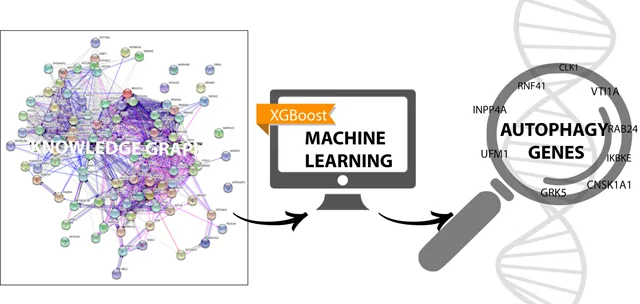

## INTRODUCTION

Autophagy (ATG) is a cytoplasmic homeostatic process defined by a suite of ATG genes conserved from yeast to man.^1^ ATG keeps a cellular complement of organelles in a functional state and removes protein aggregates, invading pathogens, and other cargo, by capturing them and delivering them to lysosomes for degradation.^1,2^ ATG is consequential for a wide array of physiological and pathological states, including neurodegenerative and other degenerative disorders, cancer, chronic inflammatory illnesses, and infectious diseases, particularly intracellular viral defense and production of infectious viral particles.^3,4^ Mechanistically, the process of ATG is governed by ATG genes, encoding the core ATG machinery with most components conserved from fungi to humans.^1^ However, recent studies have increasingly emphasized the existence of systems controlling and executing ATG in mammalian cells that are quite different from those in yeast.^5^ Among well-accepted examples, are several ATG factors absent in yeast that have been identified in organisms from *Caenorhabditis elegans* to *Homo sapiens*, including *ATG101*,^6^ *FIP200*,^7^ *VMP1*,^8^ *EPG5*,^9^ *Stx17*,^10,11^ and *TMEM41B*.^12–14^

Autophagy is a relatively new topic in biological sciences, and many aspects are still under vigorous investigation.^15,16^ There is no standardized experimental procedure to discover ATG genes in the lab. Researchers rely on accumulated cell biological information to identify genes involved.^17,18^ One of the goals of this study is to predict genes that could be potentially important in autophagy machinery but have been neglected in autophagy-related experiments. Machine learning (ML) for functional data mining could be helpful in pinpointing such genes. Because genomics provides large datasets, it is easier to delve into these with ML. Resulting gene lists would aim to inform the design relevant laboratory wet-bench studies.^19,20^

Completing the ATG puzzle requires systematic identification of all genes associated with ATG.^15^ This ongoing endeavor is boosted partly by the growing interest in the role of ATG in aging regulation^21,22^ and lifespan extension.^23^ ATG genes have been used in supervised ML models applied to aging research^24^ and are among the top features in models for predicting lifespan-extending chemicals.^25,26^ Numerous studies have used ML methods to infer gene-disease associations.^27–30^ However, the use of ML models to guide further ATG research has not been previously discussed. Here, we report the development of a specific ML model to predict “ATG dark genes,” that is, understudied proteins^31^ that may play a significant role in ATG.

## MATERIALS AND METHODS

### Developing knowledge graphs

Over 262.3 million, is unnecessary data points were extracted from 17 protein and gene-centric data sources (Table 1). They were used to develop knowledge graphs (KGs). KGs were developed by integrating data sources into formal data representation systems, with heterogeneously typed nodes and edges, which enable us to use network-based analytical algorithms. Although most of the data in Table 1 refer to human proteins, we use gene orthology relationships from *eggNOG* 4.5^32^ and *InParanoid* 8.0^33^ to merge rat (Rat Genome Database, RGD)^34^ and mouse (International Mouse Phenotype Consortium, IMPC)^35^ model organism data into a single “pseudo-protein” that enables network-based inferences for function and phenotype across organisms. The KGs are implemented as a *PostgreSQL* database for performance, convenience, and integration with existing tools. MetaPaths are rigorously defined via schema and templated in Structured Query Language (SQL). The initial prototype was primarily coded in R, which was migrated to Python for enhanced functionality and integration options, for example, visualization tools.^19^ The code and documentation are publicly accessible at https://github.com/unmtransinfo/ProteinGraphML (new Python package) and https://github.com/unmtransinfo/metap (R code). A snapshot of the currently used dataset for building KGs is available at https://unmtid-dbs.net/download/ProteinGraphML/metap-pgdump.sql.gz.

### MetaPath/XGBoost using knowledge graphs

Most biological system networks are diversified with multiple types of nodes and edges, as illustrated by the data types in Table 1. Advances in heterogeneous^51,52^ and biological system networks^53^ connection prediction have proposed and codified a new framework that considers biological system networks heterogeneity by defining type-specific node-edge routes or MetaPaths.^54^ A MetaPath^51,55^ is a path composed of a series of relations created between distinct object kinds (i.e., structural paths at the meta-level). In biology, the MetaPath method can identify various network connections between proteins/genes and specific qualities, such as phenotype or disease.^54^ We differentiate between “MetaPath” (the approach) and “MetaPaths,” which are utilized to evaluate network topology. In question are graph nodes, which can be genes or proteins, diseases or phenotypes, chemical structures or medications, and other items pertinent to modeling biological processes. “Structural pathways” represent the relationships among various entities, that is, nodes. “Paths,” or edges in topological terminology, can be expression data, pathways, LINCS genomic signatures, protein–protein interactions (PPIs), or other biologically relevant linkages.

The MetaPath (Figure 1a) approach converts the heterogeneous KG to feature vectors to run the ML algorithm; features can be categorical or continuous variables. This transformation is a type of graph embedding, which is the process of embedding information into the feature vector space. Furthermore, the KG is transformed by matching the MetaPaths to the training set for a given input query and a training set of known disease-associated genes. To construct an in-memory KG using Python library “NetworkX,” we utilized SQL queries to extract matching MetaPath entities from the Target Central Resource Database (TCRD)^56^ and transformed the SQL output into node relationships. Enumeration of all network paths along a defined MetaPath is used to obtain graph topological characteristics such as path frequencies by counting all path instances along that MetaPath. “Degree-weighted path counts” (DWPCs—see Equation 1)^53^ assign various weights to each route instance based on the number of Edges linking each node along a MetaPath. When computing DWPCs, DWPCs measure MetaPath incidence using a dampening exponent (w, set to 0.4) to downweight pathways across high-degree nodes:

(1) $$\text{DWPC}=\sum_{\text{path}\in \text{paths}}\left(\prod_{d\in D_{\text{path}}}d^{-W}\right)$$

where ∏ is the path-degree product computed by (i) collecting all edge-specific degrees along on the path $D_{path}$, where two degrees are provided by each edge; (ii) elevating each degree $\left(d\right)$ to the power of w (negative “dampening exponent”); and (iii) multiplying all exponentiated degrees to obtain ∏. DWPC is the sum of path degree products ∏.^53^ MetaPath is a formalization that allows the transformation of disparate KG data types into ML-ready input. In addition to the MetaPath-based features, a set of static features was also generated for sources invariant with disease query: Gene Tissue Expression (GTEx),^37^ LINCS,^50^ cancer cell line encyclopedia (CCLE),^36^ and HPA.^38^ These static features were independent of training set labels. Figure 1b depicts the algorithmic pathway for incorporating d into the MetaPath methodology.

To understand and predict “function” or “role in disease/phenotype,” we selected extreme gradient boosting (XGBoost),^57^ an award-winning ML algorithm that can directly be deployed onto typed MetaPaths. XGBoost classifiers have certain advantages compared to other ML algorithms: (i) fast model training and prediction and high scalability to very large datasets; (ii) variable selection is driven by model performance as opposed to random, such as that used in comparable ML algorithms; and (iii) built-in model interpretability. XGBoost outputs variable importance in projection (VIP) estimates, unlike neural networks and other deep learning methods. Using VIP, MetaPaths are sorted in decreasing order of importance. This leads to directly interpretable insights into mechanistic processes and *node* interactions that contribute to protein/gene “function” or “role in disease.” The combination of MetaPath/XGBoost (herein referred to as “MPxgb”) processes assertions/evidence chains of heterogeneous biological data types and identifies similar assertions. These determine the strength of the evidence linking a gene/protein to a disease or phenotype or, in this instance, ATG.

### Development of an ATG-specific MPxgb model

In March 2019, we extracted data from 17 protein-centric available datasets collected from the TCRD,^56^ as summarized in Table 1. Specifically for this study, we started with 288 human genes from a specialized resource, an Autophagy Database,^58^ which were processed and sorted for uniqueness. These genes were further queried for further confirmation of their ATG association using the “hsa04140” keyword in the Kyoto Encyclopedia of Genes and Genomes, KEGG^59^ Pathway, using the gene ontology (GO)^60^ term “GO:0061919”; or the UniProt^61^ keyword “autophagy,” respectively. The secondary query used to confirm the importance of specific genes in ATG resulted in *N* = 103 genes (Supporting Information). As of December 2022, we confirmed that “GO:0061919” and “hsa04140” are still valid GO and KEGG terms, as is “autophagy” in UniProt.

A separate set of OMIM genes (*N* = 3468) associated with various monogenic diseases and absent in the Autophagy Database were assumed to lack ATG involvement. We trained the MPxgb model using the 103 confirmed ATG genes as “positive” labels (class 1), that is, known to be associated with ATG, and the OMIM (*N* = 3468) monogenic disease genes as “negative” labels (class 0). To address the issue of imbalanced training data, where there were more negatives than positives, we applied a weighting strategy in our XGBoost model. Specifically, we assigned higher weights to ATG-associated genes (positive labels) using the XGBoost *scale_pos_weight* parameter, with the value set to the ratio of negative labels to positive labels. We generated 100 randomized MPxgb models using 10-fold cross-validation; the median area under the curve (AUC) for these models was 0.994 ± 0.008. As we observed a slight variance in the AUC, the model with the highest AUC (0.99993) was selected to predict novel ATG genes and is discussed here. The Excel file generated in 2019, with the complete training set (3571 genes, both positive and negative labels), is available as Table S1.

### STRING analysis

We deployed version 11.0 STRING^62^ (https://string-db.org/) to visualize PPIs networks, generated by our results based on dark ATG genes. We selected in Basic Settings: meaning of network edges: molecular actions; all active interaction sources; minimum required interaction score of 0.7 (high confidence); maximum number of interactions: no more than 50, and then in Advanced Settings interactive SVG for network display mode. We performed eight iterations with different inputs as detailed in the following section.

## RESULTS AND DISCUSSION

This paper introduces an ATG-dedicated ML model, which intends to guide the current ATG knowledge to gene sets that were previously understudied. Its primary purpose is to combine current levels of evidence and show how such evidence can be captured and modeled using a binary classifier based on the MetaPath (Figure 2) and XGBoost approach, built upon 17 different data sources (Table 1). Biologically, this model implicates a set of up to 193 previously untested genes, some of which may have a functional role in ATG.

For this model, we contrasted data for 103 ATG-associated genes with similar data for 3468 “negative” genes, extracted from 7 distinct categories of evidence: *gene and protein expression* (CCLE^36^; GTEx^37^; and the Human Protein Atlas^38^); *biological pathways and gene function* (Reactome,^39^ KEGG^59^ and GO^60^); *PPI* (STRING^63^); *protein structure and function* (InterPro^43^); *human gene-disease/phenotype associations* (ClinVar,^44^ GWAS Catalog,^45^ OMIM,^46^ UniProt Disease,^61^ and DISEASES^48^); *animal gene-phenotype associations* (mouse data from IMPC^35^ and rat data from RGD^34^ mapped to human genes via the NCBI Homology^49^ resource); and *drug-induced gene signatures from the Library of Integrated Network-Based Cellular Signatures, LINCS*,^50^ respectively. From these resources, MetaPaths were assembled, such as [protein—(member of) → STRING PPI network ← (member of)—protein—(associated with) → disease] and [protein—(expressed in) → tissue/cell line ← (associate with)—GO term]. The first biological system networks application to model new disease associations,^53^ using MetaPaths, utilized logistic regression, and ridge logistic regression. We adapted the MetaPath framework to XGBoost, a tree-boosting classification algorithm^57^ used in 17 of the 29 winning solutions in Kaggle challenge competitions.^64^ Combining XGBoost, a scalable ML algorithm capable of handling sparse data with biological system networks typed MetaPaths; we used the MPxgb approach to predict novel protein functions given a variety of (unrelated) data sources.

The methodology introduced in this work has substantial advantages over simply applying ML to an existing, static dataset. *KG* construction is informed by expertise spanning several biomedical domains, with automation tools designed for an online learning paradigm to incorporate the latest findings. Predictions presented here are not generated ex nihilo but from the rigorously aggregated findings of ATG research to date. Our methodology uses powerful ML tools to abstract that collective intelligence. This will be subject to change and improvement as the field advances.

### Model validation via receiver operating characteristics (ROC) curves

ROC curves are a standard method for evaluating and visualizing model performance with the AUC a simple measure of performance.^65^ Although, for balanced training datasets, a binary classification paradigm is appropriate, our situation is quite different, with relatively few positively labeled ATG genes, and negatives which are less certain, individually and as representatives of non-ATG genes. Thus, the validation presented does not validate our models as a classification method. Evaluated as such, given the sparsity of known genes, the specificity is poor. However, classification is not the goal. Rather, our task is to aggregate evidence to illuminate undiscovered ATG genes. The validation presented simply measures overrepresentation, or enrichment (as in gene set enrichment analysis), of known ATG genes in relation to model scores, indicating agreement and independent confirmation between two very different sources of knowledge, and the ability to improve the probability of ranking and thereby finding ATG genes relative to random, addressing an urgent need for ATG knowledge and drug target illumination.

### Model validation through variable importance selection

The top 40 features (total gain, 0.9) used to derive the best MPxgb ATG model are depicted in Figure 2 and available as Table S1. Briefly, there are 11 PPIs, with a cumulative gain of 0.445; 4 InterPro domain, IPR terms and one UniProt term (cumulative gain, 0.195); 9 GO terms (cumulative gain, 0.182); 9 LINCS terms (cumulative gain, 0.044); 3 pathway terms (cumulative gain, 0.017); and 3 expression terms (cumulative gain, 0.016), respectively. Using PPIs and structural terms provides a 64% contribution to the ATG MPxgb model; by contrast, pathway and expression terms. The presence of “autophagy” in 6 GO terms and one UniProt term provides a cumulative gain of 0.154. The related terms, “autophagy of mitochondrion”; “autophagy of nucleus”; and “autophagosome membrane,” respectively, are not present in the top 60 descriptors and, with a cumulative gain of 0.003, are not contributing to the model. This likely concerns the lack of associations between these terms and the 103 ATG-positive genes. The presence of GO and UniProt features associated with ATG could be regarded as “data leakage”^66^ as genes related to these terms, even if not present in the training set, are likely to be ATG-associated genes. However, except for the four genes that we discuss later (*VPS4A*, *RNF41*, *VTI1A*, and *RAB24*), none of the top 20 “ATG dark genes” contain GO or KEGG term annotations related to ATG. For the bottom 20 “ATG dark genes,” only 1 of them (*TMEM41B*) has ATG-related GO term annotation (Table S2).

LINCS-associated terms do not provide a significant contribution to the model. This is not surprising as the LINCS descriptors used here are derived from measuring cancer cell genomic response to drug perturbation, perhaps less relevant in ATG. Among the top 40 descriptors are 11 PPI nodes: *ATG12*, *MAPK1*, *ATG4B*, *BECN1*, *SQSTM1*, *PIK3CD*, *MARK2*, *MAPK15*, *AKT2*, *GABARAPL2*, and *TSC1*, respectively, with the top 4 (*ATG12*, *MAPK1*, *ATG4B*, and *BECN1*) accounting for 40% of the total gain. Except for *MARK2* and *MAPK15*, these form a complex network of protein interactions (https://bit.ly/2JHygyq, Figure S1). Most STRING^63^ analysis enrichment terms point to ATG (including macro-autophagy, autophagy of mitochondrion, and autophagosome assembly); phosphotransferase, and kinase activity; as well as five publications related to ATG.^67–71^ We note that STRING v11.0 was used in this analysis.^62^

Of the two outlier PPI nodes, *MARK2* and *MAPK15*, *MAPK15* is the part of the ULK complex.^72^ It stimulates ATG by interacting with ATG8 family proteins.^73^ The involvement of *MARK2* in ATG is less clear.^74^ When combined with the 103 ATG-model input genes, a complex regulatory network enriched in the same ATG terms is observed (https://bit.ly/2Z2SF60, Figure S2), with 3 additional KEGG pathways: *hsa04211: longevity regulating pathway*, *hsa04150: mTOR signaling pathway*, and *hsa04068: FoxO signaling pathway*, respectively. The top 5 publications also relate to ATG.^15,68,75–77^ Based on the STRING analysis of MPxgb-selected PPI nodes, we conclude that these descriptors are relevant for ATG. The MPxgb variable selection of IPR domains and GO terms is also related to genes that play a relevant role in ATG (see also Supporting Information). We conclude that the ATG MPxgb model discussed herein is significantly enriched in ATG-related terms and PPI nodes and bears relevance for guiding future ATG research with respect to potentially novel gene annotation for ATG involvement.

### Model output: quantifying knowledge

We examined the top 251 predicted genes, ranked by the predicted probability of association with ATG. Among these, 9 were present in the initial set of 288 Autophagy Database genes; another 34 genes are annotated in the Autophagy Database expanded set (see Supporting Information). A total of 43 genes predicted by the ATG MPxgb model were retrieved from Autophagy Database. Another 15 genes could be found by performing additional queries, such as “autophagy pathway” in PathCards.^78^ Thus, 58 of the top 251 genes, or 23.1%, appear to have confirmed (or strongly suspected) an association with ATG. However, 193 of these top 251 genes were not associated with ATG, despite efforts to consult various literature-based resources and online databases (which include Autophagy Database, PathCards, KEGG, GO, UniProt, and TCRD)—in 2019.^79^ Although *Z*-score values are above 2 for these 251 genes, predicted probability drops from 0.967 (row 105, GABARAPL3) to 0.08464 (row 355, CHMP7). Even though GABARAPL3 and CHMP7 have corroborating annotations concerning their role in ATG, this dramatic drop in probability makes us question the validity of exploring genes with values below 0.0846. XGBoost may appear to output a gradient, but values close to zero are much more likely to lack associations with ATG.

Given that most of the top 40 ATG MPxgb variables and 23% of the top 251 genes (including the gene ranked 246, *ARFGEF1*—present in Autophagy Database), we posit that this ML model bears relevance in the study of ATG and suggest that some of these 193 genes may represent ATG “dark genes.” We also examined the “target development level” (TDL), a knowledge-based classification for human proteins^31^ that can be used to explore the dark genome.^80^ In brief, “Tclin” are proteins via which approved drugs act (i.e., mode-of-action drug targets); “Tchem” are proteins known to bind small molecules with high potency; “Tbio” are proteins with well-studied biology, having a fractional publication count above 5^81^ or well-annotated OMIM (disease) phenotypes; and “Tdark” are understudied proteins that do not meet criteria for the above three categories, respectively. The TDL count for the training set (103 ATG-associated genes), the test set (top 251 predicted genes), and the “ATG dark genes?” subset (193 genes) is summarized in Table 2. As estimated by this knowledge classification system, it can be concluded that most of the predicted genes, specifically for the ATG dark genes, are either Tbio or Tdark. Indeed, nearly 30.6% of the “ATG dark genes” are classified as “Tdark,” which suggests that a significant portion of these genes is understudied.

### Model output: newly predicted genes

For the sake of brevity, we focus the remainder of this discussion on the top 40 predicted genes, which are outlined in Table 3. Of these, 15 genes are already in Autophagy Database (*GABARAPL3, ULK3, EIF2AK2, RRAGD, RAB1A, VPS39, CSNK1G2, SNX1, CSNK2A2, PRKAB1, RAB9A, PRKCI, MAP3K14, EXOC4*, and *CALCOCO2*). *RMDN1* interacts with beclin 1, a known regulator of ATG^82^; another gene, SLK, escorts *VPS4B* which itself is involved in ATG^83^; *OXSR1* is upstream of AMPK, a known ATG regulator^84^; whereas *MTMR3* and *BNIP3L* are ATG-annotated in PathCards.

In 2019,^79^ we suggested that 20 of the top 40 genes may represent novel assertions for autophagy: *MAP1LC3B2*, *TAOK3*, *NEK6*, *CLK1*, *STK35*, *NLK*, *VPS4A*, *RNF41*, *CLK3*, *VTI1A*, *RAB24*, *UBALD1*, *S100A6*, *PRKD2*, *NRBP1*, *CSNK1A1*, *RBM18*, *IKBKE*, *GRK5*, and *TMEM167A*, respectively. With 6 exceptions (*TAOK3*, *CLK1*, *CLK3*, *UBALD1*, *RBM18*, and *TMEM167A*), the remaining 14 genes form a complex network when combined with the 11 PPI nodes discussed in the Variable Importance section (https://bit.ly/2Yc5EoZ, Figure 3). As previously mentioned, the STRING analysis enrichment terms point to ATG (including autophagy, macro-autophagy, and autophagosome assembly); protein serine/threonine kinase activity; KEGG pathway terms “hsa04140: autophagy—animal,” “hsa05167: Kaposi’s sarcoma-associated herpesvirus infection,” “hsa05160: hepatitis C” and “hsa04152: AMPK signaling pathway” as well as multiple publications related to ATG.^68,69,71,75,85–88^

As many of the potentially novel 20 genes out of the top 40 are kinases, we used X2K Web (eXpression2Kinases)^89^ to perform a transcription factor enrichment analysis (TFEA).^90^ This type of analysis predicts which transcription factors (TFs) are most likely to regulate the expression of these 20 genes. The ranked list of predicted TFs is visually summarized in Figure 4. The top-ranked 13 TFs, selected to ensure that each of the top 20 predicted ATG genes is represented at least once, are as follows: *RUNX1, TCF7L2, ELF1, TAF1, GATA1, FOXP2, FOXA1, SPI1, CHD1, SRF, FOXA2, ZNF384*, and *BRCA1*, respectively.

When combining the 11 PPI node genes selected by the ATG MPxgb model with the 13 TFEA-selected genes, the top potentially novel ATG genes form a complex STRING-based network (https://bit.ly/2YeOffr, Figure S3) that leaves six genes out (*NRBP1, CLK1, CLK3, UBALD1, RBM18*, and *TMEM167A*). In addition to GO terms related to ATG, the STRING analysis enrichment terms include “TF binding” and “membrane-bounded organelle”; the UniProt keyword “phosphoprotein”; the KEGG pathway terms *hsa03022: basal TFs*, *hsa04137: mitophagy—animal* and *hsa05168: herpes simplex infection*, as well as several publications related to ATG.^67,68,71,85,91–93^ Although the TFEA step does appear to include STRING enrichment terms associated with TFs, it also provides additional data elements supporting the role of some proteins left out of the STRING-based PPI network: *UBALD1* might be regulated by 10 of the 13 TFs, *TAOK3* and *CLK1* by 5, and *TMEM167A* by 4 TFs, whereas *NRBP1* and *RBM18* are regulated by 2. Both this and the prior STRING network highlight the relationship between some ATG-related genes and viral (*H. simplex*, *hepatitis C*) infection. This is not surprising as ATG is an antiviral defense mechanism that interacts with intracellular viral particles.^94^

### Model output: validation and data leakage

To validate MPxgb predictions, we explored the top 20 predicted genes for literature reports related to ATG because we suggested these as “ATG dark genes” in 2019.^79^ All the gene aliases were obtained from the NCBI Gene database^95^ to ensure query completeness. The literature search was conducted in PubMed (https://pubmed.ncbi.nlm.nih.gov/). The results are shown in Table 4. For 9 out of 20, we could identify studies that report them as ATG genes.

While analyzing this model, we were concerned about “data leakage,” given the presence of GO and KEGG terms related to ATG (or variants thereof) in the Feature set. Specifically, ATG associations for the “ATG dark genes” set would obfuscate and render the ML predictions questionable. Table S2 contains hyperlinks to the GO terms and pathway annotations for these top 20 genes, as indexed^56^ in Pharos 2023. None of these top 20 genes have a KEGG term associated with ATG. Four ATG dark genes validated by recent publications, *CLK1, CSNK1A1, IKBKE*, and *GRK5*, also lack ATG-related GO annotations. In the case of *MAP1LC3B2*, we found two terms in Pharos 2023: “autophagosome assembly” (GO:0000045) and “autophagy of mitochondrion” (GO:0000422), respectively. However, references to these two functions were published^96,97^ after 2019. Hence, our ATG prediction for this gene was true and novel.

For *VPS4A*, the term “macro-autophagy” (GO:0016236) references a “non-traceable author statement” (GO terminology) annotated by ParkinsonsUK-UCL^117^; Table 1 in this 2010 paper^117^ lists “autophagy” as a function for *VPS4A* and discusses the role of the ESCRT (endosomal sorting complexes required for transport) machinery in ATG; although *VPS4A* is part of ESCRT, there is no further discussion of the *VPS4*-ATG association. For *VTI1A*, the term “autophagy” (GO:0061919), “inferred from mutant phenotype” (GO terminology) was annotated by ParkinsonsUK-UCL^118^; this 2013 paper^118^ references *VTI1A* as an *STX13* binding partner in the autophagosome. For the other two genes, *RNF41* and *RAB24*, the GO:0061919 term annotation was inferred from UniProt-KW “by similarity” (as referenced in their respective UniProt entries as of December 2022). These annotations were not present in the 2019 version of UniProt (as indexed in version 5.4 of TCRD, data not shown). Based on current evidence, it seems prudent to conclude that data leakage informed predictions for 2 (*VPS4A, VTI1A*) out of the 9 recently confirmed genes. However, the KG used to train the 2019 ML model did not “leak” ATG-related terms for 7 of the 9 literature-validated genes.

When analyzing MPxgb gene predictions for a model trained for Alzheimer’s disease^19^ (AD), we found that, in addition to 5 of the top 20 MPxgb AD-predicted genes, 3 of the bottom 10 genes were also altered in a manner relevant to AD pathology. We, therefore, noted that our MPxgb AD model did “not distinguish an AD-association gradient,” and thus, “the bottom” (lowest probability) predicted genes do not necessarily lack AD association.’’ For this reason, we extended our validation efforts to the 20 bottom ranked “ATG dark genes.” The results, obtained via a process similar to the previous step (Table 4 above), are shown in Table 5.

With the same concern about “data leakage,” GO term annotations associated with ATG were traced for 3 of the 20 lowest ranking “ATG dark genes” recently reported as ATG genes. *TMEM41B*, a recently highly studied ATG gene, has been previously studied on its association with ATG machinery,^132^ as well as ATG-related GO term annotation, “autophagosome assembly” (GO:0000045).^56^ The other two, *UFM1* and *INPP4A*, would not be influenced by “data leakage.”^56^ In other words, the MPxgb model built on the 2019 KG model^79^ did not “leak” ATG-related terms for 2 of the 3 confirmed genes from the lowest ranking set.

The STRING network created using the top 20 MPxgb predicted genes (Table 4) shows a minimally connected network (https://bit.ly/3hKEMrP, Figure S4) with “purine ribonucleotide binding” (GO:0035539, *N* = 13) and “protein phosphorylation” (GO:0006468, *N* = 11) as the main enrichment GO terms and the Pfam domain “protein tyrosine kinase” (PF07714, *N* = 11) in the STRING “analysis” tab. Most genes are singletons, with only three pairs (*CLK1*-*CLK3*, *RAB24*-*VPS4A*, and *IKBKE*-*RNF41*) connected. The STRING network created using the bottom 20 MPxgb predicted genes (Table 5) shows an almost absent network (https://bit.ly/3PJEspQ, Figure S5) with practically no enrichment. Terms such as “glucuronosyltransferase activity” (GO:0015020, *N* = 3) and the Pfam domain “UDP-glucuronosyl and UDP-glucosyl transferase” (PF00201, *N* = 3) in the STRING “analysis” tab illustrate the lack of pattern in this specific subset. All genes except one pair (*UFM1*-*MOCS3*) are singletons. Limiting the network to the top 9 literature-validated genes (Table 4) plus the 9 relevant PPI STRING nodes selected via MPxgb modeling (discussed above) clearly leads to “autophagy” as the dominant term in STRING Analysis (https://bit.ly/3YLaPJ4, Figure S6), with 3 of the top 9 nodes (*CLK1*, *VTI1A*, and *GRK5*) as singletons. Repeating this analysis with the bottom 3 literature-validated genes (Table 5) plus the same 9 relevant PPI STRING nodes selected via MPxgb modeling (discussed above) also leads to “autophagy” as the dominant term in STRING Analysis (https://bit.ly/3WEaAO6, Figure S7), but 2 of the bottom 3 nodes (*TMEM41B* and *INPP4A*) remain singletons. According to STRING enrichment analyses, the top 20 and the bottom 20 gene sets do not share similarities: The “top 9” genes are more likely to be relevant to a network of ATG-associated proteins than the “bottom 3” genes.

The top 9 predicted genes do not represent absolute ATG-associated ranking nor are the bottom 3 genes least likely to be ATG-associated. The difference between top- and bottom-predicted genes appears to be in their similarity network: Top genes are more likely to point in a similar direction (e.g., “purine ribonucleotide binding” and “protein tyrosine kinase”), whereas the bottom list appears less organized, such as STRING enrichment analyses. Although “top” versus “bottom” probability does not provide a definitive verdict regarding ATG association or lack thereof, it does appear that top-ranked genes are more likely to participate in this complex process. We believe that a more specific ML model, perhaps based on a different KG, may be required to build an accurate picture of ATG. As this ML model is based on similarity networks (MetaPaths), its ranked list of ATG-associated genes is imperfect. As written elsewhere,^19^ improving this ML model would require a “ground truth” (confirmed true positives and true negatives) training set. If our negative gene set actually contains genes involved in ATG, the features from those genes would lead to a false negative in our processing.

How might this information empower future wet-bench experiments pursuing molecular mechanisms of ATG function toward pharmacological interventions? It might be argued that there are too many possibilities for wet-bench approaches that are typically reductionist, testing one candidate gene at a time. For such focused experiments, we propose investigators choose from among those fewer ATG genes with AD associations that are fewer in number, as such disease relationships suggest functional relevance. Alternatively, those genes with the most ATG features would also be more likely to yield results in wet-bench experiments. However, molecular discovery has moved beyond candidate gene testing.^133^ It could be possible to design a high-throughput experiment that tests all 193 dark ATG genes at once using multi-well plates and probes for each predicted dark ATG gene, either siRNA for knockdown or transfections to increase expression, with a readout measuring ATG processed fragments.^134^ For HSV1 functional relevance, Vero cells infected with HSV1 could be subjected to siRNA knockdown for each dark ATG and viral production measured.^4,94^

## CONCLUSIONS

In this paper, we introduced an ATG-dedicated ML model, which we intend to provide as guidance for extending current ATG knowledge to explore genes that previously have not been evaluated for their role in ATG. In developing this ATG-specific MPxgb model, we started with 288 genes associated with ATG from the Autophagy Database,^58^ upon which we set additional filters (association with GO, KEGG, and UniProt terms) to increase confidence in the remaining 103 ATG-associated “positive” genes. For non-ATG-associated “negative” genes, 3468 genes were selected that are associated with monogenic diseases in OMIM and not present in the Autophagy Database. We used XGBoost, a binary classifier, combined with the MetaPath approach, which was deployed upon 17 sources representing 7 distinct categories. The ATG MPxgb model implicates up to 193 previously untested genes that have a predicted functional role in ATG. This fulfills our primary motivation: *To provide the scientific community with an ML-ready KG and an ML-derived ATG gene list, as summarized by the training set, combined with a truly blind prediction set of 193 dark ATG genes*. Through literature queries, we found that 7 of the top 20 predicted “dark genes” and 3 of the bottom 20 genes were subsequently validated as genes involved in ATG in 20 newly published experimental reports. By collating different datasets and weaving different sets of information together, we highlight understudied ATG dark genes. This work will inspire researchers investigating ATG to prioritize certain genes out of larger Autophagy Database. These recent reports show the potential strength of our ML model to extend knowledge within functional genomic libraries. We considered shortcomings of our MPxgb ATG model including: (i) imbalance of training sets (overcome with a weighting strategy); (ii) “data leakage” (check the presence of “autophagy” GO terms in the MetaPath models); (iii) ranking genes by probability should be interpreted with caution; (iv) lack of a “ground truth” list of true negatives, which could lead to missing some ATG genes. By disclosing this list of putative genes, we aim to encourage further experimental testing. Our methodology could ultimately also prove powerful for the discovery of “dark genes” for other functional activities within cells. All gene sets mentioned throughout this paper are available in the Supporting Information section. Future validation steps may include integrating chemical–protein annotation resources, using Pharos^56^ and Chem-Prot,^135^ calculating SHAP values,^136^ and using a semantic model to evaluate druggability via the drug target ontology.^137^

## Supplementary Material

Suppl Table 1.xlsx

Suppl Table 2.xlsx

Supplementary Material Fig 1

SUPPORTING INFORMATION

Additional supporting information can be found online in the Supporting Information section at the end of this article.

## Figures and Tables

**FIGURE 1:**
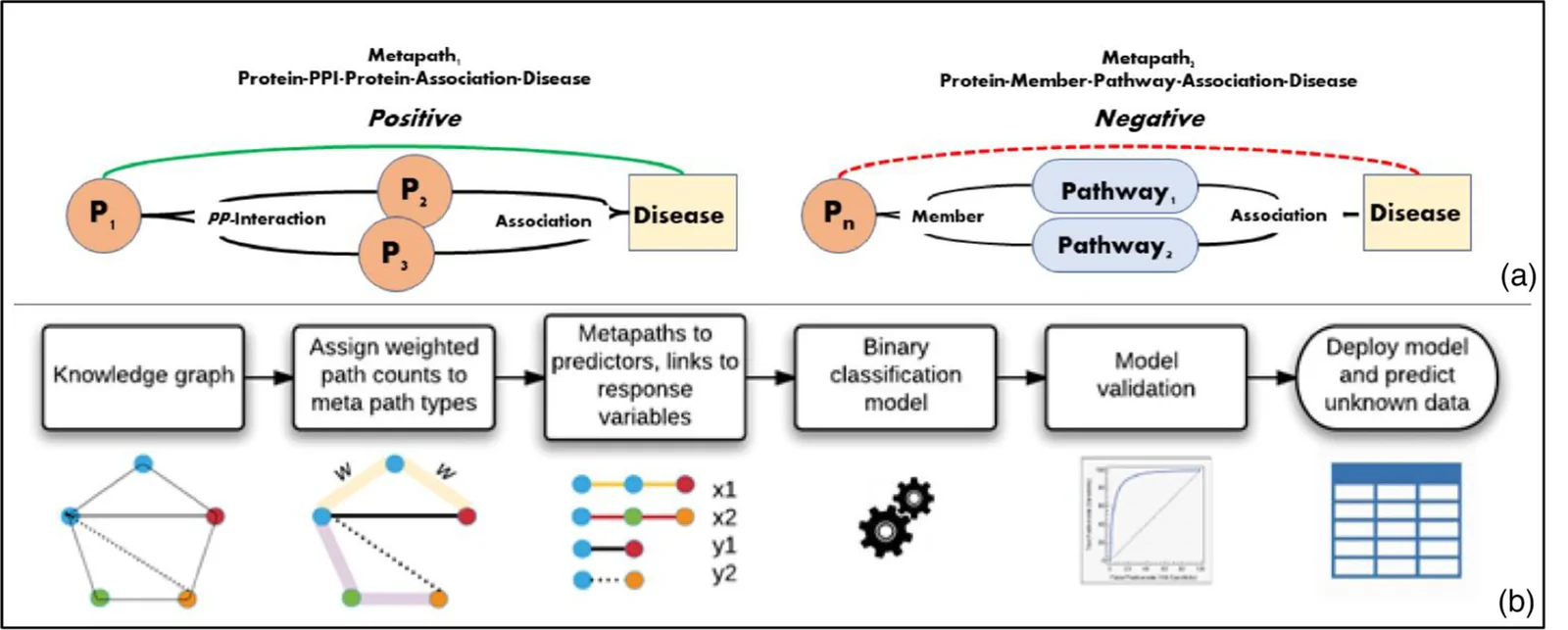
(a) A MetaPath illustration, (b) analytical algorithms workflow for ranking target gene lists. After obtaining nodes and edges to build the knowledge graph, paths or edges would be weighted to get the model.

**FIGURE 2:**
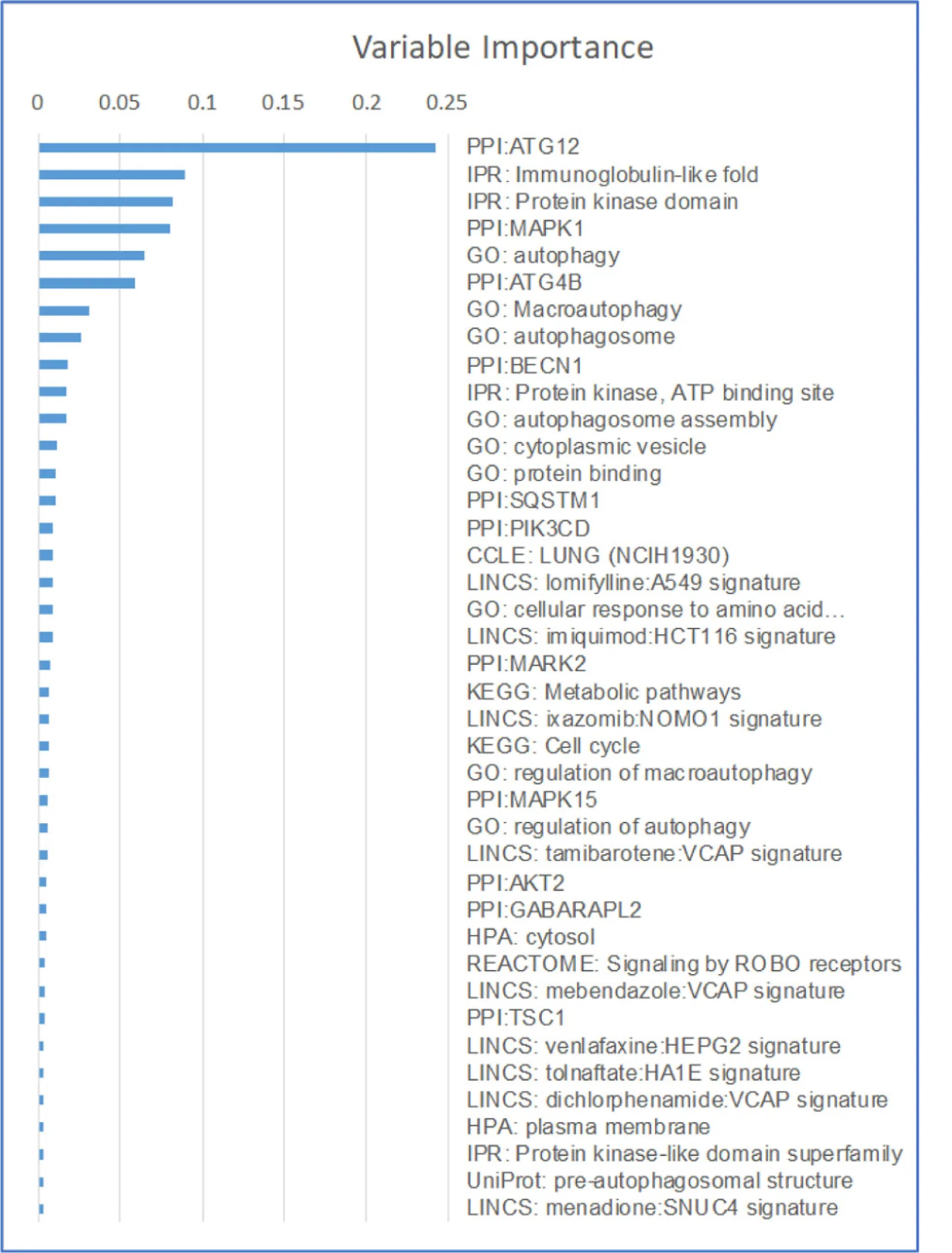
Top 40 variables, selected by the ATG MPxgb model, in decreasing order of importance. GO, gene ontology term; HPA, human protein atlas; IPR, InterPro domain; PPI, protein–protein interaction nodes. (See text for additional details.)

**FIGURE 3:**
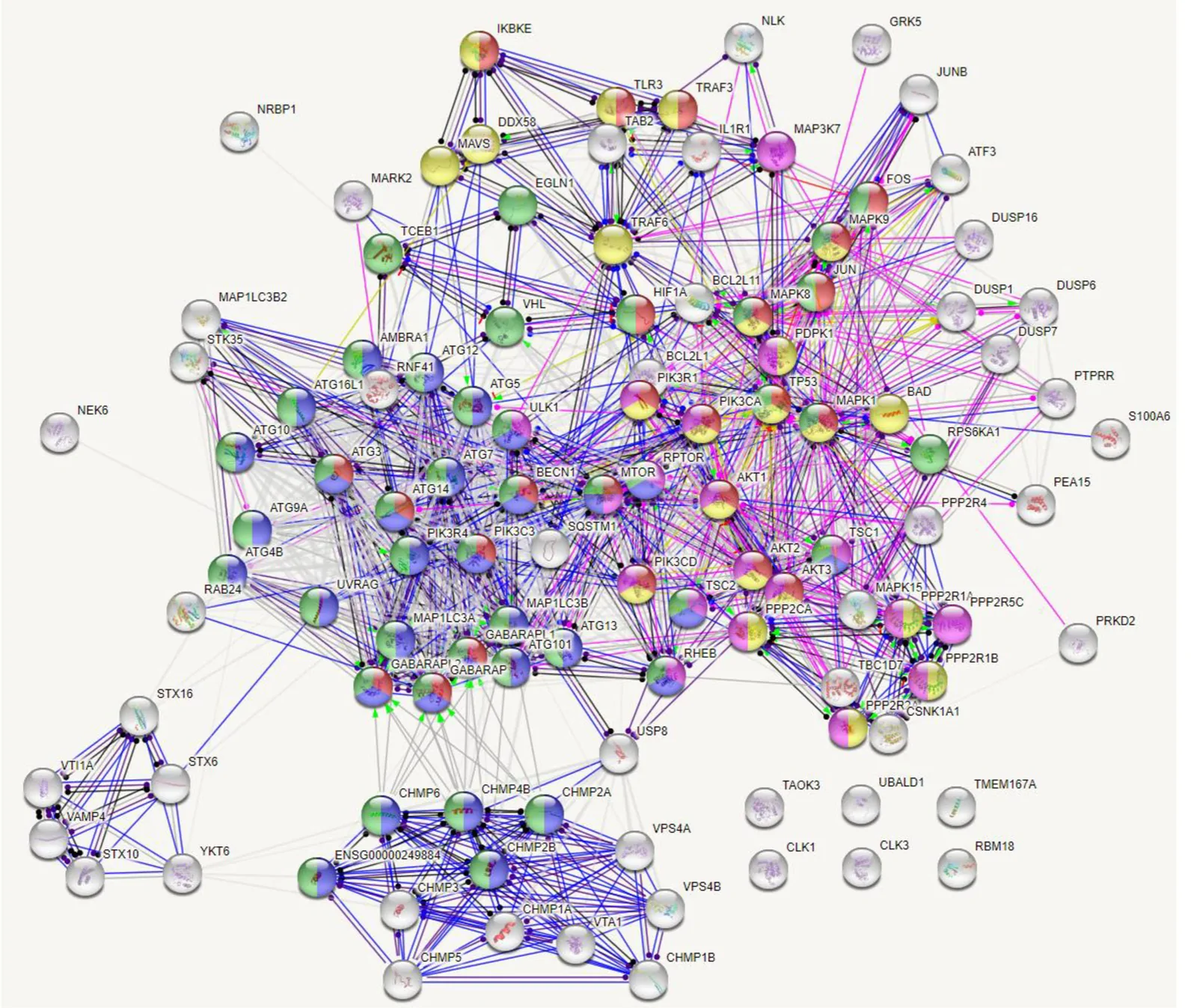
Visual presentation of the protein–protein interaction (PPI) of the top 14 genes plus 11 relevant PPI nodes selected via MPxgb as the dominant terms in STRING analysis. Six “exceptions” are shown as singletons at the bottom right.

**FIGURE 4:**
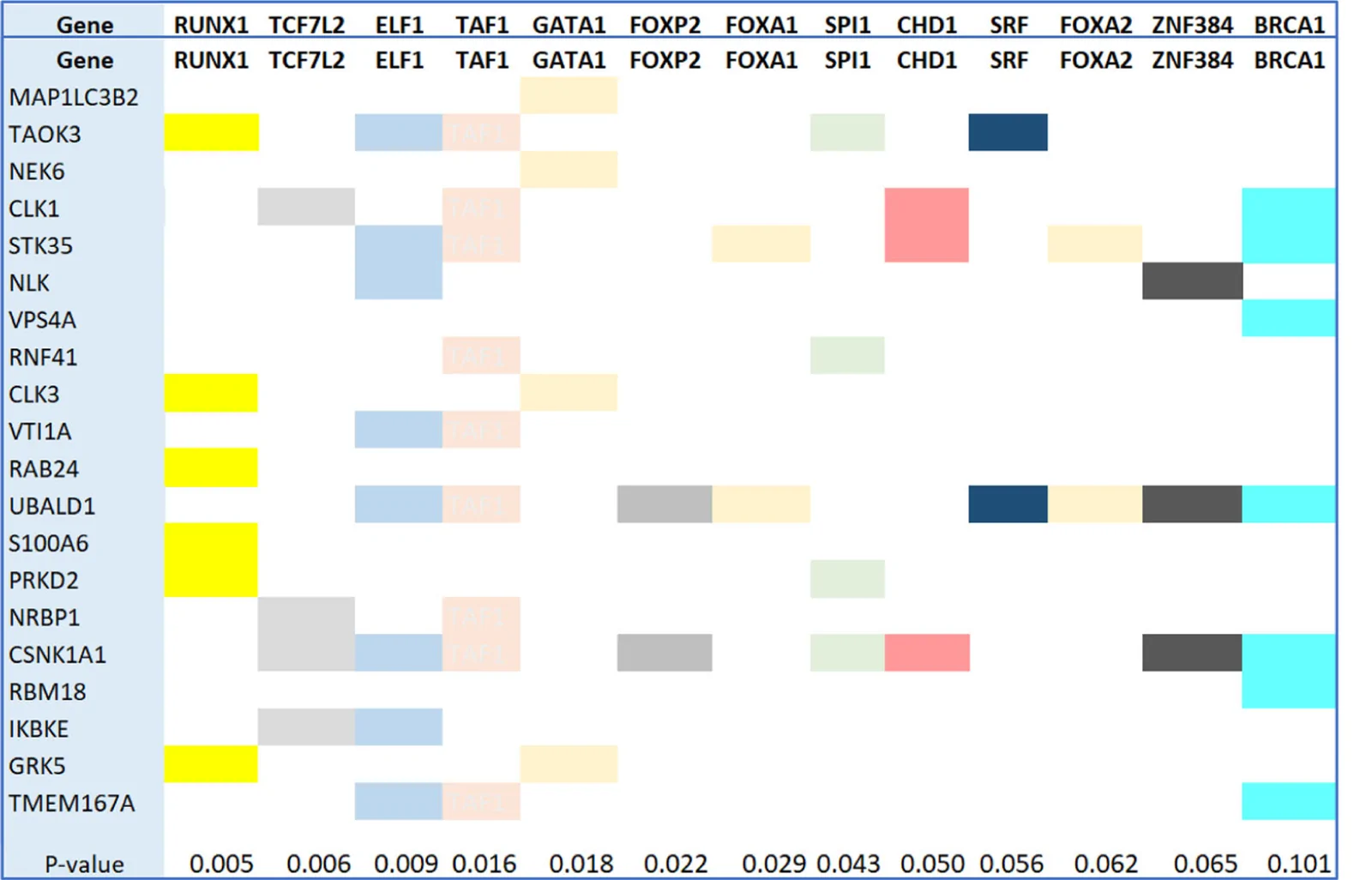
A visual summary of the transcription factor enrichment analysis for the top 20 predicted genes ATG dark genes. Thirteen transcription factors are shown in reverse order of *p*-value ranking. (See text for additional details.)

**TABLE 1:** Data sources used to inform the development of machine learning (ML)-ready knowledge graphs (as of 2019).

Data source	Data type	Data points

CCLE^36^	Gene expression	19,006,134
GTEx^37^	Gene expression	2612,227
HPA^38^	Gene and protein expression	949,199
Reactome^39^	Biological pathways	303,681
KEGG^40^	Biological pathways	27,683
STRING^41^	Protein–protein interactions	5080,023
GO^42^	Biological pathways and gene function	434,317
InterPro^43^	Protein structure and function	467,163
ClinVar^44^	Human gene—disease/phenotype associations	881,357
GWAS Catalog^45^	Gene—disease/phenotype associations	54,360
OMIM^46^	Human gene—disease/phenotype associations	25,557
UniProt disease^47^	Human gene—disease/phenotype associations	5365
DISEASES^48^	Gene—disease associations from text mining	44,829
NCBI Homology^49^	Homology mapping of human/mouse/rat genes	70,922
IMPC^35^	Mouse gene—phenotype associations	2153,999
RGD^34^	Rat gene—phenotype associations	117,606
LINCS^50^	Drug-induced gene signatures	230,111,315

Abbreviations: CCLE, cancer cell line encyclopedia; GO, gene ontology; GTEx, Gene Tissue Expression; IMPC, International Mouse Phenotype Consortium; KEGG, Kyoto Encyclopedia of Genes and Genomes.

**TABLE 2:** Target development level distribution for the ATG MPxgb model sets.

Set	Tclin	Tchem	Tbio	Tdark

Training	10	32	60	1
Test	0	52	137	62
ATG dark gene?	0	37	97	59

**TABLE 3:** List of top 40 genes from the ATG MPxgb model.

UniProt accession	HGCN symbol	Protein name	Pred.prob	TDL	Autophagy information

Q9BY60	GABARAPL3	Gamma-aminobutyric acid receptor-associated protein-like 3	0.967	Tdark	Present in Autophagy Database (288 set)
Q6PHR2	ULK3	Serine/threonine-protein kinase ULK3	0.927	Tchem	Present in Autophagy Database (expanded set)
Q96DB5	RMDN1	Regulator of microtubule dynamics protein 1	0.917	Tdark	Beclin interacting/STRING-DB
P19525	EIF2AK2	Interferon-induced, double-stranded RNA-activated protein kinase	0.910	Tchem	Present in Autophagy Database (expanded set)
Q9NQL2	RRAGD	Ras-related GTP-binding protein D	0.841	Tbio	Present in Autophagy Database (expanded set)
O95747	OXSR1	Serine/threonine-protein kinase OSR1	0.818	Tbio	Upstream of AMPK
Q9H2G2	SLK	STE20-like serine/threonine-protein kinase	0.770	Tchem	escort VPS4B
Q13615	MTMR3	Myotubularin-related protein 3	0.749	Tbio	Autophagy pathway PathCards|Autophagy animal PathCards
P62820	RAB1A	Ras-related protein Rab-1A	0.731	Tbio	Present in Autophagy Database (expanded set)
A6NCE7	MAP1LC3B2	Microtubule-associated proteins 1A/1B light chain 3 beta 2	0.713	Tdark	ATG dark gene?
Q9H2K8	TAOK3	Serine/threonine-protein kinase TAO3	0.709	Tchem	ATG dark gene?
Q9HC98	NEK6	Serine/threonine-protein kinase Nek6	0.703	Tchem	ATG dark gene?
Q96JC1	VPS39	Vam6/Vps39-like protein	0.695	Tbio	Present in Autophagy Database (expanded set)
P49759	CLK1	Dual specificity protein kinase CLK1	0.686	Tchem	ATG dark gene?
O60238	BNIP3L	BCL2/adenovirus E1B 19 kDa protein-interacting protein 3-like	0.683	Tbio	Autophagy pathway PathCards
Q8TDR2	STK35	Serine/threonine-protein kinase 35	0.676	Tchem	ATG dark gene?
P78368	CSNK1G2	Casein kinase I isoform gamma-2	0.675	Tchem	Present in Autophagy Database (expanded set)
Q9UBE8	NLK	Serine/threonine-protein kinase NLK	0.652	Tchem	ATG dark gene?
Q9UN37	VPS4A	Vacuolar protein sorting-associated protein 4A	0.651	Tbio	ATG dark gene?
Q9H4P4	RNF41	E3 ubiquitin-protein ligase NRDP1	0.630	Tbio	ATG dark gene?
Q13596	SNX1	Sorting nexin-1	0.619	Tbio	Present in Autophagy Database (288 set)
P49761	CLK3	Dual specificity protein kinase CLK3	0.585	Tchem	ATG dark gene?
Q96AJ9	VTI1A	Vesicle transport through interaction with t-SNAREs homolog 1A	0.585	Tbio	ATG dark gene?
P19784	CSNK2A2	Casein kinase II subunit alpha’	0.577	Tchem	Present in Autophagy Database (expanded set)
Q9Y478	PRKAB1	5′-AMP-activated protein kinase subunit beta-1	0.576	Tchem	Present in Autophagy Database (expanded set)
P51151	RAB9A	Ras-related protein Rab-9A	0.518	Tbio	Present in Autophagy Database (expanded set)
Q969Q5	RAB24	Ras-related protein Rab-24	0.517	Tbio	ATG dark gene?
P41743	PRKCI	Protein kinase C iota type	0.512	Tchem	Present in Autophagy Database (expanded set)
Q8TB05	UBALD1	UBA-like domain-containing protein 1	0.496	Tdark	ATG dark gene?
P06703	S100A6	Protein S100-A6	0.468	Tbio	ATG dark gene?
Q9BZL6	PRKD2	Serine/threonine-protein kinase D2	0.462	Tchem	ATG dark gene?
Q99558	MAP3K14	Mitogen-activated protein kinase kinase kinase 14	0.453	Tchem	Present in Autophagy Database (expanded set)
Q9UHY1	NRBP1	Nuclear receptor-binding protein	0.437	Tbio	ATG dark gene?
P48729	CSNK1A1	Casein kinase I isoform alpha	0.436	Tchem	ATG dark gene?
Q96H35	RBM18	Probable RNA-binding protein 18	0.430	Tdark	ATG dark gene?
Q96A65	EXOC4	Exocyst complex component 4	0.415	Tbio	Present in Autophagy Database (expanded set)
Q14164	IKBKE	Inhibitor of nuclear factor kappa-B kinase subunit epsilon	0.402	Tchem	ATG dark gene?
P34947	GRK5	G protein-coupled receptor kinase 5	0.395	Tchem	ATG dark gene?
Q8TBQ9	TMEM167A	Protein kish-A	0.394	Tdark	ATG dark gene?
Q13137	CALCOCO2	Calcium-binding and coiled-coil domain-containing protein 2	0.394	Tbio	Present in Autophagy Database (expanded set)

*Note*: HUGO Gene Nomenclature Committee. Pred.prob—predicted probability, as derived from the model.

Abbreviation: TDL, target development level.

**TABLE 4:** Validation of the predictions made by the machine learning (ML) model for the top 20 predicted genes.

Gene name	Gene aliases	Recently reported as autophagy gene?

MAP1LC3B2	ATG8G	**Yes** ^96–99^
TAOK3	DPK, JIK, MAP3K18, hKFC-A	No
NEK6	SID6-1512	No
CLK1	CLK, CLK/STY, STY	**Yes** ^100,101^
STK35	CLIK1, STK35L1	No
NLK	*NA*	No
VPS4A	CIMDAG, SKD1, SKD1A, SKD2, VPS4, VPS4-1	**Yes** ^102–105^
RNF41	FLRF, NRDP1, SBBI03	**Yes** ^106^
CLK3	PHCLK3; PHCLK3/152	No
VTI1A	*NA*	**Yes** ^107^
RAB24	*NA*	**Yes** ^108–111^
UBALD1	FAM100A, PP11303	No
S100A6	2A9, 5B10, CABP, CACY, PRA, S10A6	No
PRKD2	HSPC187, PKD2, nPKC-D2	No
NRBP1	BCON3, MADM, MUDPNP, NRBP	No
CSNK1A1	CK1, CK1a, CKIa, HEL-S-77p, HLCDGP1, PRO2975	**Yes** ^112^
RBM18	*NA*	No
IKBKE	IKK-E, IKK-i, IKKE, IKKI	**Yes** ^113–115^
GRK5	FP2025, GPRK5	**Yes** ^116^
TMEM167A	*NA*	No

**TABLE 5:** Validation of the predictions made by the machine learning (ML) model for the 20 lowest ranking predicted genes.

Gene name	Gene aliases	Recently reported as autophagy gene?

HEATR9	C17orf6	No
MROH5	*NA*	No
PPP4R3C	FLFL3PP, SMEK3P, smk1, PPP4R3C	No
UGT1A5	UDPGT, UDPGT 1–5, UGT1E	No
UGT1A8	GNT1, UDPGT, UDPGT 1–1, UDPGT 1–8, UGT-1A, UGT-1H, UGT1, UGT1-01, UGT1-08, UGT1.1, UGT1.8, UGT1A, UGT1A1S, UGT1H, hUG-BR1, UGT1A8	No
UGT1A3	UDPGT, UDPGT 1–3, UGT-1C, UGT1-03, UGT1.3S, UGT1C, UGT1A3	No
MOCS3	UBA4	No
SEMA4B	SEMAC, SemC	No
TOR2A	TORP1	No
CARMIL2	CARMIL2b, IMD58, LRRC16C, RLTPR	No
DENND5B	*NA*	No
ZGRF1	C4orf21	No
UBTD2	DCUBP	No
TMEM41B	*NA*	Yes^119–127^
UFM1	BM-002, C13orf20, HLD14	Yes^128–130^
INPP4A	INPP4, TVAS1	Yes^131^
DDX42	DDX42P, RHELP, RNAHP, SF3B8, SF3b125	No
ARMC10	PNAS-112, PNAS112, PSEC0198, SVH	No
LDHC	CT32, LDH3, LDHX	No
AGAP6	AGAP-6, CTGLF3	No

## Data Availability

The data that support the findings of this study are openly available in repositories as described in this submitted manuscript. The genes resulting from the machine learning are listed in Tables S1–S2.

## Key points:

- A knowledge-graph based machine learning model was designed for predicting unknown autophagy genes via mining functional genomic data.
- Literature search validated predicted genes.
- Our machine learning models could be generalized and applied to other genomiclibraries to uncover dark genes for various functions.
