# Supplementary material for: Autophagy dark genes: Can we find them with machine learning?
Source: Nat Sci (Weinh). Author manuscript; Available in PMC 2026 Oct 8. (PMC13644537; doi:10.1002/ntls.20220067)
Supplement: Supplementary file 1 [file NIHMS2133995-supplement-Supplementary_Material_Fig_1.docx]

**Guide for the nodes and edges:**

**Nodes:**

Network nodes represent proteins,

*splice isoforms or post-translational modifications are collapsed, i.e. each node represents all the proteins produced by a single, protein-coding gene locus.*

Node Color

*colored nodes:
query proteins and first shell of interactors*

*white nodes:
second shell of interactors*

Node Content

*empty nodes:
proteins of unknown 3D structure*

*filled nodes:
some 3D structure is known or predicted.*

**Edges:**

Edges represent protein-protein associations,

*associations are meant to be specific and meaningful, i.e. proteins jointly contribute to a shared function; this does not necessarily mean they are physically binding each other.*

Action Types

*activation*

*inhibition*

*binding*

*catalysis*

*phenotype*

*posttranslational modification*

*reaction*

*transcriptional regulation*

Action effects

*positive*

*negative*

*unspecified*

**Figure S1**. Visual presentation of the 11 Protein-protein interactions (descriptors) obtained from STRING analysis.

**Figure S2.** Visual presentation of the interactions of PPI node genes selected by the ATG MPxgb model with the 103 ATG-model input genes.

**Figure S3.** Visual presentation of the interactions of the 11 PPI node genes selected by the ATG MPxgb model with the 13 TFEA-selected genes, the top potentially novel ATG genes.

**Figure S4.** Visual presentation of the top 20 MPxgb predicted genes from Table 4, with “purine ribonucleotide binding” (GO:0035539, N=13) and “protein phosphorylation” (GO:0006468, N=11) as the main enrichment GO terms and the Pfam domain “protein tyrosine kinase” (PF07714, N=11)

**Figure S5.** Visual presentation of the bottom 20 MPxgb predicted genes from Table 4, with “purine ribonucleotide binding” (GO:0035539, N=13) and “protein phosphorylation” (GO:0006468, N=11) as the main enrichment GO terms and the Pfam domain “protein tyrosine kinase” (PF07714, N=11)

**Figure S6.** Visual presentation of the top 9 literature-validated genes from **Table 4** plus the 9 relevant PPI STRING nodes selected via MPxgb modeling (discussed above)

**Figure S7**. Visual presentation of the protein-protein interaction of 3 bottom literature-validated genes plus 9 relevant PPI nodes selected via MPxgb as the dominant terms in STRING analysis.
